# Characterisation of novel microRNAs in the Black flying fox (*Pteropus alecto*) by deep sequencing

**DOI:** 10.1186/1471-2164-15-682

**Published:** 2014-08-15

**Authors:** Christopher Cowled, Cameron R Stewart, Vladimir A Likic, Marc R Friedländer, Mary Tachedjian, Kristie A Jenkins, Mark L Tizard, Pauline Cottee, Glenn A Marsh, Peng Zhou, Michelle L Baker, Andrew G Bean, Lin-fa Wang

**Affiliations:** CSIRO Australian Animal Health Laboratory, 5 Portarlington Rd, Geelong East, Kragujevac, Victoria 3220 Australia; Bio21 Molecular Science and Biotechnology Institute, Melbourne, Australia; Centre for Genomic Regulation (CRG) and Universitat Pompeu Fabra (UPF), Barcelona, Spain; Program in Emerging Infectious Diseases, Duke-NUS Graduate Medical School, Singapore, 169857 Singapore

**Keywords:** Bats, Chiroptera, Pteropus alecto, MicroRNA, Non-coding RNA, Transcriptome

## Abstract

**Background:**

Bats are a major source of new and emerging viral diseases. Despite the fact that bats carry and shed highly pathogenic viruses including Ebola, Nipah and SARS, they rarely display clinical symptoms of infection. Host factors influencing viral replication are poorly understood in bats and are likely to include both pre- and post-transcriptional regulatory mechanisms. MicroRNAs are a major mechanism of post-transcriptional gene regulation, however very little is known about them in bats.

**Results:**

This study describes 399 microRNAs identified by deep sequencing of small RNA isolated from tissues of the Black flying fox, *Pteropus alecto*, a confirmed natural reservoir of the human pathogens *Hendra virus* and *Australian bat lyssavirus*. Of the microRNAs identified, more than 100 are unique amongst vertebrates, including a subset containing mutations in critical seed regions. Clusters of rapidly-evolving microRNAs were identified, as well as microRNAs predicted to target genes involved in antiviral immunity, the DNA damage response, apoptosis and autophagy. Closer inspection of the predicted targets for several highly supported novel miRNA candidates suggests putative roles in host-virus interaction.

**Conclusions:**

MicroRNAs are likely to play major roles in regulating virus-host interaction in bats, via dampening of inflammatory responses (limiting the effects of immunopathology), and directly limiting the extent of viral replication, either through restricting the availability of essential factors or by controlling apoptosis. Characterisation of the bat microRNA repertoire is an essential step towards understanding transcriptional regulation during viral infection, and will assist in the identification of mechanisms that enable bats to act as natural virus reservoirs. This in turn will facilitate the development of antiviral strategies for use in humans and other species.

**Electronic supplementary material:**

The online version of this article (doi:10.1186/1471-2164-15-682) contains supplementary material, which is available to authorized users.

## Background

More than 20% of all mammalian species are bats, making them an extraordinarily important and successful group from an evolutionary perspective [1]. Bats are unique amongst mammals for their ability to fly, and possess notable traits such as long life expectancy in proportion to body size [2]. Many species of bats exhibit exotic traits including echolocation and hibernation, and bats are an important part of the ecosystem via plant pollination and insect control [3]. Notoriously, bats are also reservoir hosts for a large number of zoonotic viruses [4]. Understanding the mechanisms by which bats co-exist with and seemingly tolerate viruses that are deadly in humans and other mammals has implications for human health, and may facilitate development of new antiviral strategies.

One aspect of the bat-virus relationship that has not been investigated in detail is the role of host gene regulation, in particular the role of microRNAs (miRNAs). MiRNAs are essential regulators of eukaryotic gene expression [5] and include elements required for viral replication [6]. MiRNA biogenesis is a multistep process in which primary-miRNA transcripts (pri-miRNA) are cleaved into precursor-miRNA (pre-miRNA) by the nuclear RNase III DROSHA and then further processed in the cytoplasm by DICER1 to produce mature miRNAs averaging 22 nt in length [5]. This process produces transcripts from both arms of the precursor, whereas the loop and flanking sequences are destroyed. This information is exploited by miRNA-finding algorithms such as miRDeep2 for the identification of miRNA-like sequences in small RNA transcriptome data.

Mature miRNAs have been found in over 150 species of plants, animals and viruses [7, 8]. While no *P. alecto* miRNAs have yet been reported, recent studies identified miRNAs in the Little brown bat *Myotis lucifugus* [9–12], the Big brown bat, *Eptesicus fuscus* [13], and the Jamaican flying fox, *Artebius jamaicensis* [14]. Additionally, bat genomes within the Ensembl database (*P. vampyrus* and *M. lucifugus*) feature miRNA annotations based on homology to the human genome.

In this study, the small RNA transcriptome of the Black flying fox (*P. alecto*) was sequenced from a pooled tissue sample. MiRDeep2 was used to identify conserved and novel miRNAs, and a variety of methods were employed to assess those predictions. Target prediction and annotation enrichment analysis were undertaken to identify miRNAs with putative roles in pathways that are relevant to virus-host interaction. These results will enable fundamental insights into miRNA-mediated gene regulation in bats, and are an important step towards determining the molecular basis of bats’ role as virus reservoirs.

## Results

### Identification of *P. alecto*miRNAs

**Figure 1:**
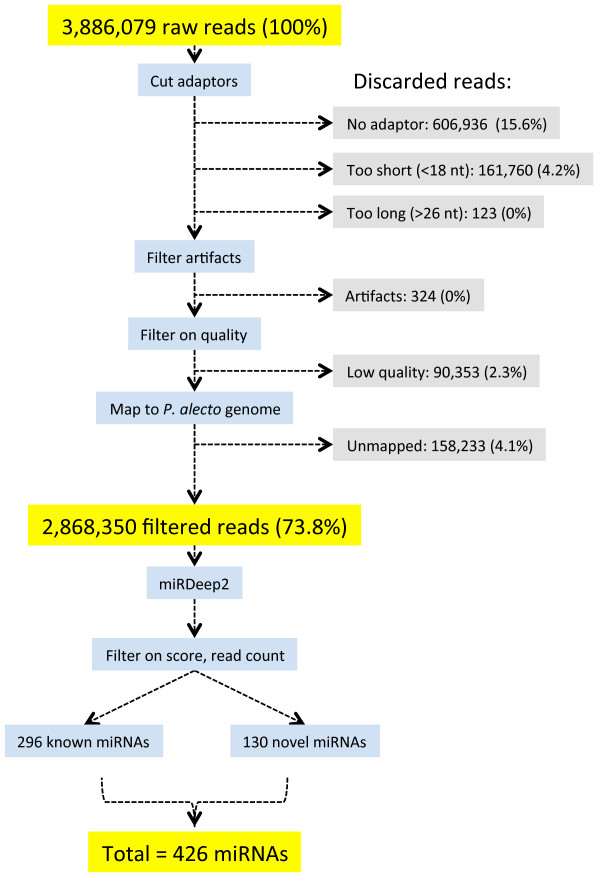
**Overview of the miRNA discovery pipeline.** Unsuitable reads were discarded at each stage of preprocessing. High quality reads were subjected to miRDeep2 analysis, followed by further culling based on novelty, score and read depth. A total of 426 *P. alecto* miRNAs passed the initial filtering stages. A further 27 candidates were later culled from the analysis.

**Figure 2:**
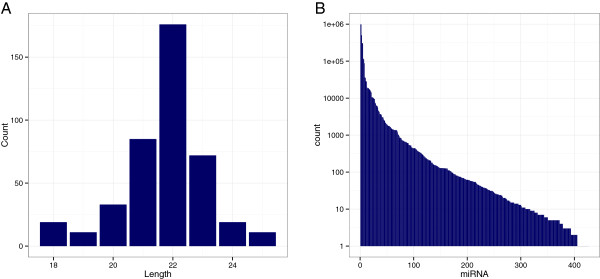
**Size and frequency of** ***P. alecto*** **mature miRNAs. (A)** Length distribution of *P. alecto* mature miRNAs. **(B)** Read-count distribution of mature miRNAs (trimmed + filtered + mapped reads only). Drawn in log_10_ scale.

Illumina deep-sequencing of small RNA from pooled *P. alecto* tissues yielded a total of 3,886,079 reads, each 36 nucleotides in length, which have been submitted to the Sequence Read Archive (SRA) under BioProject accession number PRJNA210946. Figure 1 illustrates the analysis pipeline used to identify miRNAs. Approximately 30% of the raw reads were discarded during data filtering, mainly due to absence of the 3’ adapter (15.6%). Data quality was examined before and after trimming and filtering (Additional file 1: Figure S1). MiRNAs were then identified using miRDeep2. As no *P. alecto* miRNAs were known prior to this undertaking, miRDeep2 was provided with a large set of learning information in the form of all known vertebrate miRNAs from a recent release of miRBase (version 19). This resulted in identification of 426 distinct miRNA precursors, from which 551 different mature and star transcripts were detected (Figure 2, Additional file 2: Table S1). While 361 (85%) of the precursors mapped to a unique locus, the remaining 65 consisted of 31 overlapping pairs plus an overlapping triplet. For 27 of these pairs, the presence of star reads for one member only and/or large differences in miRDeep2 scores indicated the likely precursor (Additional file 3: Table S2). The lower scoring precursor in each of these cases was excluded from further analysis. Six overlapping pairs remained unresolved, either due to an absence of star reads or because mature and star sequences were palindromic, thus the set of *P. alecto* miRNAs in this dataset consists of 399 candidates.

MiRDeep2 flagged four putative miRNAs as possible tRNA/rRNA. To incorporate the latest knowledge of non-coding (nc) RNA, we compared predicted *P. alecto* miRNA precursors with all known ncRNA in a recent version of RFAM (version 11.0) (Additional file 4: Table S3). A total of 222 *P. alecto* miRNAs were supported by RFAM hits to known miRNAs. A further nineteen exhibited similarity to other types of ncRNA. Hits to ncRNAs other than miRNAs do not necessarily preclude candidates from being true miRNAs, however they may be less reliable than other candidates.

### Homology to miRNAs in other species

**Figure 3:**
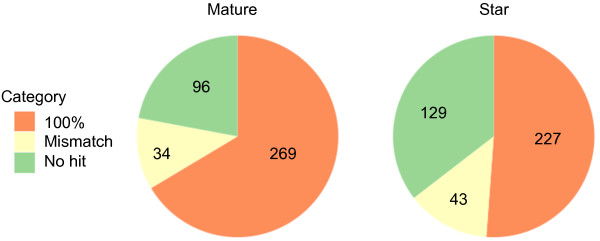
**Homology of bat miRNAs to vertebrate mature miRNAs in miRBase.** *P. alecto* mature and star miRNAs were compared to a database of all vertebrate miRNAs extracted from miRBase (version 20) using BLASTN. 100% identity indicates that no internal gaps or mismatches were present in alignments between *P. alecto* miRNAs and their respective top BLAST hits. Minor length variations were allowed in this category, but the alignments were end-anchored to prevent mismatches at the termini. MiRNAs in the mismatch category had internal mismatches or indels relative to their top BLAST hits, whereas miRNAs in the no-hit category had no convincing BLAST hits. Search parameters are described in detail in the materials and methods.

Bat miRNAs were compared to the following collections of reference miRNA sequence data: MiRBase version 20 (all vertebrates); Biggar et al. [9]; Platt et al. [13]; Ensembl (*P. vampyrus*, *M. lucifugus*). Of the 399 *P. alecto* miRNAs examined, a total of 233 mature sequences and 145 star sequences were 100% identical to mature vertebrate entries in miRBase. *P. alecto* miRNAs were then compared to the reference data using BLASTN (Figure 3, Additional file 5: Table S4). To allow for minor length variations, all mature and star BLAST searches were filtered such that the ends of each alignment were allowed to overhang but were not allowed to mismatch. This revealed an additional 36 *P. alecto* mature miRNAs that had hits to miRBase 20 vertebrates with 100% internal identity but one or more over- hanging or under-hanging ends. A further 34 mature *P. alecto* miRNAs had high-scoring hits to miRBase 20 vertebrates but contained internal mismatches or gaps, while 96 *P. alecto* mature miRNAs had no reliable hits against miRBase 20 vertebrates at all. This process was repeated using mature, star and precursor sequences against each of the reference datasets, producing hits for a further 30 *P. alecto* miRNAs. Of the remaining 66 *P. alecto* miRNAs that had no BLAST hits at all, 35 had miRDeep2 scores ≥ 1 and three had miRDeep2 scores ≥ 30.

Notably, two *P. alecto* precursors (pal-can-280 and pal-can-392) had large numbers of hits to *P. vampyrus* precursors (38 and 40 hits, respectively), in each case representing a variety of similar but different sequences rather than one single repeated sequence. In contrast, a single *P. alecto* precursor (pal-can-136) had a similarly high number of hits in *M. lucifugus*, however this appeared to be due to low-complexity composition of the precursor (GU repeats) as opposed to genuine homology.

### MiRNAs in introns and clusters

Many miRNAs are located within the introns of protein-coding genes [15]. We identified 98 miRNAs physically located within introns of annotated bat genes, while three (pal-can-011, pal-can-306, pal-can-346) were fully overlapping with coding exons (Additional file 6: Table S5). Of these, pal-can-346 (miR-1306) is located within an exon of DGCR8, a protein involved in miRNA biogenesis in humans [16]. Five miRNAs overlapped with exon boundaries and could potentially be regulated by splicing events [17].

Many miRNAs are arranged in local clusters on chromosomes [18]. The *P. alecto* genome currently consists of contigs and scaffolds that are not yet mapped to individual chromosomes, however with an N_50_ size of 15.8 Mb [19], many scaffolds are easily large enough to contain such clusters. MiRNA clusters were located by identifying miRNAs spaced less than 5000 bp apart. miRNA pairs located < 60 bp apart were shown to be overlapping as described above. Such pairs were treated as a single entity and were only considered part of a cluster if they grouped with at least one other non-overlapping miRNA. A total of 42 clusters comprising 140 miRNA genes were identified according to these criteria (Additional file 7: Table S6).

The largest identified bat miRNA cluster contained 33 miRNAs and is homologous to the recognised *DLK1*-*DIO3* cluster on human chromosome 14, known to be involved in disease pathogenesis [20]. Three novel bat miRNAs were located within this cluster, including one (pal-can-276) that returned BLAST hits to miR-541 but had three mismatches relative to other vertebrates, including two unique differences in the seed region. For the two other novel bat miRNAs within this cluster (pal-can-411 and pal-can-252), the mature sequences produced non-identical BLAST hits to miR-376 and miR-377, respectively, however the star sequences were 100% identical to miR-376 and miR-377 star sequences, respectively.

**Figure 4:**
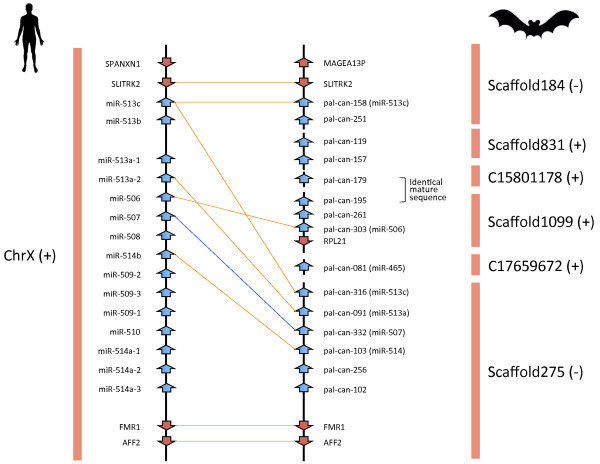
**Proposed clustering of** ***P. alecto*** **miRNAs corresponding to the human ChrX miR-506:514 cluster.** Protein-coding genes are denoted by red arrows, miRNAs by blue arrows. *P. alecto* mature miRNAs that yielded BLAST hits to known vertebrate miRNAs are connected to their human counterpart by colored lines indicating whether they shared 100% sequence identity (blue) or were non-identical (orange).

A total of 22 novel *P. alecto* miRNAs were located within 13 clusters, and five clusters contained only novel miRNAs. Two such clusters each contained three novel miRNAs that all scored highly by miRDeep2. The first of these consisted of pal-can-102, pal-can-256 and pal-can-103. The closest nearby genes, *FMR1* and *AFF2*, are located a distance of 0.5-1.5 Mb from the miRNA cluster. In the human genome, *FMR1* and *AFF2* are located on the X chromosome, adjacent to two recognised miRNA clusters (miR-888:892 and miR-506:514). One of the three bat miRNAs in this cluster (pal-can-103) returned a non-identical BLAST hit to dog miR-514, while the other two returned only low-quality hits. The second cluster consisted of pal-can-195, pal-can-261 and pal-can-303. One of these (pal-can-303) returned a BLAST hit to miR-506, while the other two returned only low-quality BLAST hits (however these included miR-465, which is also located on the X chromosome in humans and a member of an equivalent miRNA cluster in rodents). Thus the two novel-only clusters appear to represent parts of the same cluster split over multiple scaffolds. Based on homology, nine additional miRNAs were identified that may also belong to this cluster. A proposed arrangement is shown in Figure 4. Overall, 15 bat miRNAs may represent members of a cluster homologous to the human X-chromosomal miR-506:514 cluster. Only one of these (pal-can-332) shares 100% mature sequence identity with a known vertebrate miRNA (miR-507), while the remaining 14 are unique to *P. alecto*. Alignment of the individual sequences indicated that while BLAST was unable to identify homologues for more than half of these miRNAs, a degree of similarity amongst them was clearly evident (Additional file 8: Figure S2).

### Seed sequence analysis

The nucleotide sequence corresponding to bases 2–7 of a mature miRNA (the seed region) is the most important region for determining target specificity [21]. The seed sequences of *P. alecto* miRNAs were assessed (Additional file 9: Table S7). The 399 bat miRNAs had 273 different seed sequences with 63 seed sequences appearing more than once. The most frequently occurring seeds were GAGGUA (let-7), which appeared 12 times and CUGGAC (mir-378), which appeared 9 times. Compared to miRBase vertebrate mature miRNAs, 40 bat miRNAs (two pairs plus 17 singletons) appeared to have novel seed sequences. A potential weakness of this approach, however, is that if a miRNA has an incorrectly predicted start site, then the predicted seed sequence will also be incorrect. Therefore, only miRNAs that aligned from position #1 in both the bat mature sequence and its top BLAST hit were considered reliable enough to determine true seed sequence. Of the 49 miRNAs with non-identical top BLAST hits, 30 aligned from position #1, however only one of these (pal-can-316) had a seed sequence that was unique amongst miRBase vertebrates.

**Figure 5:**
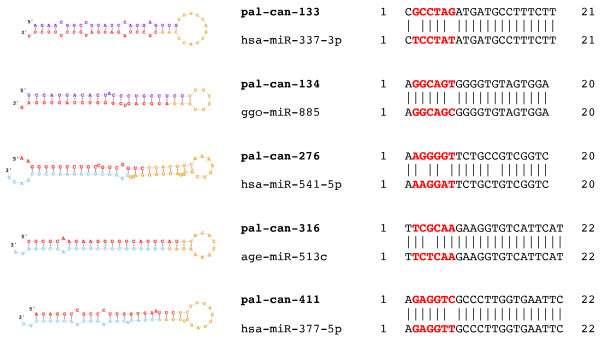
***P. alecto*** **miRNAs with novel seed sequences.** Proposed stem-loop structures of five *P. alecto* miRNAs with novel seed sequences are shown on the left, based on the minimum free energy confirmations determined by Vienna RNAfold during miRDeep2 processing. Observed mature sequences are drawn in red, predicted loop regions in yellow, observed star sequences in light blue and predicted star sequences in purple. Alignments between *P. alecto* miRNAs and their respective top BLAST hits are shown on the right with the seed regions highlighted in bold.

Given the high probability that at least one unrelated miRBase vertebrate miRNA will match any given seed sequence by chance (the odds are approximately 50:50), further steps were taken to identify bat miRNAs with novel seed sequences. MiRNAs that had non-identical top BLAST hits and also aligned from position #1 in both query and target were individually examined. Of these, five were identified as having one or more unique differences in the seed sequence relative to their top BLAST hit. To confirm these observations, mature and star miRNAs were compared against all of their BLAST hits (not just the top hit), to evaluate whether the observed seed changes were truly unique in these bat miRNAs relative to their counterparts in other vertebrates. The sequences, predicted secondary structures, and top-hit alignments of the five novel-seed miRNAs are illustrated in Figure 5.

Three of the five novel-seed miRNAs (pal-can-276, pal-can-133 and pal-can-316) had both mature and star sequences that were unique to *P. alecto* in the sense that they were not shared by any of the BLAST hits to these sequences. Two of these (pal-can-276 and pal-can-133) had seed sequences (AGGGGU and GCCUAG) that were shared by single vertebrate entries in miRBase (gga-miR-1761 and hsa-miR-3135a, respectively), yet they did not resemble those miRNAs in any other respect. In contrast, pal-can-316 seed sequence (UCGCAA) was completely unique amongst vertebrates, in the sense that it was not shared by any vertebrate miRNA, including unrelated sequences. These three miRNAs had mature/star read counts of 162/0, 36/8 and 10/0, respectively.

The two remaining *P. alecto* miRNAs (pal-can-411 and pal-can-134) had mature seed sequences distinct from those of their BLAST hits, however their star sequences matched with 100% identity to miRBase entries. Of these, pal-can-134 had mature/star read counts of 31/1; however only a single mature read was detected for pal-can-411.

### Ranking of novel candidate miRNAs

In addition to the bat miRNAs with confirmed novel seed sequences, a shortlist of the most promising novel miRNA candidates was constructed by narrowing the master list by excluding conserved miRNAs and those with low support. The following were excluded: (1) Putative miRNAs with 100%-identity BLAST hit (either mature or star) to a miRBase vertebrate entry, (2) Putative miRNAs with a miRDeep2 score below 1.0, (3) Putative miRNAs with a read count below 10, (4) Putative miRNAs that overlapped with another miRNA and either scored substantially less than the overlapping partner or could not be differentiated from it based on score, (5) Putative miRNAs with a non-miRNA hit in RFAM. This left 30 miRNAs, considered the most promising novel candidates for further study (Table 1).

**Table 1:** **Top 30 novel miRNAs in** ***P. alecto***

miRNA	miRDeep2 score	Mature reads	Loop reads	Star reads	Mature sequence	Top BLAST hit
pal-can-034	1273.2	2480	0	11	augaccuaugaaucgacagaca	cgr-miR-215-5p
pal-can-081	145.7	267	0	10	accugugcccuucugaguagc	rno-miR-465-3p
pal-can-091	91.6	130	0	41	aauggcaccuuucugaguagu	ppy-miR-513a-2-3p
pal-can-101	69.3	111	0	23	ugcugcucagggacggggcga	
pal-can-102	69.2	131	0	3	ugcuagggcuagagagcgagugc	
pal-can-103	61.3	106	0	6	ugauugacagcuuugagagugg	cfa-miR-514
pal-can-119	37.1	33	0	38	gaccuaagcccuucugaguau	
pal-can-125	30.1	45	0	5	caacucuaaggggcaucauuca	
pal-can-133	23.5	36	0	8	cgccuagaugaugccuuucuu	ppy-miR-337-3p
pal-can-140	15.3	11	5	12	aaaugguacccuagugacuaca	rno-miR-224-3p
pal-can-157	6.6	11	0	1	uaacaggcauuucugagguga	
pal-can-187	5.5	81	0	0	uuuccggcuuagugggugugu	eca-miR-1180
pal-can-179	5.5	38	0	4	uacucagaaggggccagguuac	
pal-can-195	5.4	38	0	4	uacucagaaggggccagguuac	
pal-can-227	4.9	25	0	0	aucucgguggaaccucca	
pal-can-231	4.8	44	0	0	gagagaucagaggugcagagu	bta-miR-6529
pal-can-245	4.5	10	0	0	uugcagcugccgggagugauuu	eca-miR-1301
pal-can-247	4.3	10	0	0	aggggcagcaugguguagcag	
pal-can-249	4.3	50	0	0	uagguaguuucuuguuguuggg	ssc-miR-196b
pal-can-254	3.8	267	0	0	cagaagaguagauugauuggu	
pal-can-257	2.9	69	0	0	agagguaaaaauuugauuuga	bta-miR-6119-5p
pal-can-263	2.5	43	0	0	aggaauguaaagaagcaugu	
pal-can-271	2.1	69	0	0	acaucaagacuaggcauacacug	
pal-can-276	1.8	162	0	0	aagggguucugccgucgguc	oar-miR-541-5p
pal-can-278	1.8	12	0	0	aucucgcuggggccucca	
pal-can-280	1.7	25	0	0	ucaagucccuguucgggcgcca	mmu-miR-5097
pal-can-290	1.5	23	0	0	ugggggagagaacagguagaca	
pal-can-303	1.2	14	0	0	aauuugggccuuucugaguaga	age-miR-506
pal-can-307	1	15	0	0	agccuugugacugacgaucggaca	
pal-can-316	0.9	10	0	0	uucgcaagaaggugucauucau	age-miR-513c

### Target prediction

It has been recognised that 3’ UTRs are the preferred location for miRNA binding sites in mRNA targets [5]. Target prediction was performed for the 399 mature *P. alecto* miRNAs using the target scanning algorithm miRanda and a database of 3’ UTRs derived from *P. alecto* transcriptome data. To avoid potential artefacts, excessively long 3’ UTRs (those more than 2500 nt in length) were excluded, resulting in a search space representing 6021 different *P. alecto* genes corresponding to 5899 different human genes (the remaining 122 are bat-specific gene duplications). The median number of predicted gene targets per miRNA was 77, while the median number of predicted miRNA hits per target gene was 7, which in some cases included multiple hits from an individual miRNA.

The highest-scoring miR:Target prediction was pal-can-170:*USP36*, which had 10 binding sites and a cumulative minimum free energy (E_sum) of −217.80. Other high scoring miR:Target predictions were pal-can-421:*DNMBP* (six binding sites, E_sum = −152.27); and pal-can-207/pal-can-098:*ZNF768* (six binding sites each, E_sum = −143.30), and pal-can-290:*RAPGEF1* (six binding sites, E_sum = −138.79). The highest-scoring miR:Target predictions for individual sites were pal-can-194:*ALG3* with a minimum free energy (E) of −50.51; and pal-can-300:*FANCI* (E = −45.27).

Amongst the most important miRNA targets are transcription factors, since these can extend a miRNA’s regulatory influence to multiple secondary targets. Of the ~1400 transcription factors currently recognised within the human genome [22], 325 were present within the *P. alecto* 3’ UTR database, including factors of immune-relevance such as interferon regulatory factors (*IRF1*, *IRF3*, *IRF8*) and NF-κB (*RELA*, *RELB*, *NFKB1*). MiR: Target predictions involving transcription factors are listed in Additional file 10: Table S8. Predicted targets of the novel bat miRNA pal-can-276 included the transcription factor *ZFAT*, involved in apoptosis and cell survival, as well as a number of genes with significant roles in apoptosis and host defence (Table 2).

**Table 2:** **Selected gene target predictions for novel** ***P. alecto*** **miRNA pal-can-276 (miR-541)**

Pteropus alecto gene	Gene ID	Gene name	Minimum free energy (Best hit)	3’ UTR length	Hit positions
PAL_GLEAN_10012524	*USP20*	ubiquitin specific peptidase 20	−28.73	1592	1189
PAL_GLEAN_10020514	*DDIT4*	DNA-damage-inducible transcript 4	−28.62	881	725
PAL_GLEAN_10016798	*CXXC5*	CXXC finger 5	−25.01	578	246,390
PAL_GLEAN_10009170	*USP19*	ubiquitin specific peptidase 19	−23.6	570	153
PAL_GLEAN_10002258	*POLM*	polymerase (DNA directed), mu	−23.07	2124	127
PAL_GLEAN_10012978	*TNFRSF1B*	tumor necrosis factor receptor superfamily, member 1B	−22.97	1595	155
PAL_GLEAN_10025525	*LRRC32*	leucine rich repeat containing 32	−21.93	2398	2193
PAL_GLEAN_10012819	*LRRFIP2*	leucine rich repeat (in FLII) interacting protein 2	−21.91	1906	1405
PAL_GLEAN_10006697	*CRLF1*	cytokine receptor-like factor 1	−21.56	754	651
PAL_GLEAN_10015148	*IL28RA*	interleukin 28 receptor, alpha (interferon, lambda receptor)	−21.08	1404	1334
PAL_GLEAN_10014403	*ATG9A*	ATG9 autophagy related 9 homolog A (*S. cerevisiae*)	−20.74	1437	443
PAL_GLEAN_10020540	*AIFM2*	apoptosis-inducing factor, mitochondrion-associated, 2	−20.45	2358	832
PAL_GLEAN_10010342	*ZFAT*	zinc finger and AT hook domain containing	−20.09	679	164

Categorical enrichment analysis based on Gene Ontology (GO) and KEGG pathway annotations were performed using the lists of predicted target genes for each miRNA (Additional file 11: Table S9, Additional file 12: Table S10). Ranked by Bonferroni-corrected p-values, the most significantly enriched GO terms were GO:0006357: “regulation of transcription from RNA polymerase II promoter” (pal-can-247) and GO:0000278: “mitotic cell cycle” (pal-can-379); GO:0006325: “chromatin organization” (pal-can-354). The most significantly enriched KEGG pathways were hsa04912: GnRH signalling pathway (pal-can-085); hsa04060: Cytokine-cytokine receptor interaction (pal-can-088); hsa04012: ErbB signalling pathway (pal-can-170); and hsa03030: DNA replication (pal-can-300).

## Discussion

Bats have long held special significance in the contexts of ecology and mammalian evolution, but have only recently been recognised as a major source of emerging infectious diseases [4]. To date, *P. alecto* miRNAs have not been studied yet are likely to play significant roles in important cellular functions including immunity, apoptosis, the cell cycle, inflammation, and DNA repair. In this study, 399 putative miRNAs were identified in the small RNA transcriptome of the Black flying fox, *P. alecto*, of which 269 had mature sequences with 100% identity to known vertebrate miRNAs in miRBase and 34 had homologs in miRBase but contained unique differences. The remaining 96 had no clear homology to known miRNAs, yet many were predicted with high confidence and appear to be genuine novel bat miRNAs.

The number of miRNAs identified in *P. alecto* falls broadly within the range observed for other mammals. For the 38 vertebrate species used for comparative analysis in this study, the number of miRBase records ranged from 1 to 1881. Different studies have used different tissues, different quantities of data, different methods and different parameters for identifying miRNAs, making direct comparisons difficult. In two somewhat comparable studies, miRDeep2 was used to identify 399 miRNAs in the pig intestine, including 354 with a miRDeep2 score above −3 [23], while *in silico* analysis of the horse genome identified a total of 406 miRNAs [24]. Studies in other species of bats have reported varying numbers of precursors, the highest being 762, identified using miRanalyzer on ~20 million reads, with further confirmation provided by > 200 million additional reads [13]. Another recent paper detected 887 mature and star miRNAs corresponding to 568 precursors in the mouse transcriptome, however this was achieved using almost 90 million Illumina reads [25], more than 20x the data volume used in the present study. From these comparisons it can be concluded that the number of miRNAs observed in the bat transcriptome can be considered fairly typical. For 196 of the 399 bat miRNAs, reads corresponding to both mature and star sequences were detected, further increasing the likelihood that these miRNAs result from specific miRNA biogenesis.

There is, as yet, no universally accepted procedure for identifying novel miRNAs in high-throughput sequencing data. In our study, the major factor determining the final number of miRNAs was the miRDeep2 score. As may be expected, lowering the cut-off score involves a trade-off between sensitivity (fewer false negatives) and specificity (more false positives). A cut-off score ≥ 1 provides a typical starting point for miRNA identification; however many highly-conserved miRNAs which are very likely to be real do not meet this stringent cut-off. If the cut-off score is lowered, however, the number of highly improbable candidates rapidly increases. Relying on a single, stringent cut-off point comes at a significant cost of missing many genuine miRNAs. We concluded that within our data, miRDeep2 score and read depth were the factors that best enabled miRNA identification. We chose a model that accepted all hits to known miRNAs (regardless of score), novel hits with scores ≥ 1 and read depth ≥ 2 (typical hits), and novel hits with scores ≥ −5 and read depth ≥ 5 (outliers). Following the initial screening, a further 27 candidates were culled because they overlapped higher-scoring candidates, leaving a total of 399 candidates under consideration. This model, in our opinion, reflected a better balance between sensitivity and specificity than a single parameter cut-off model. In support of our decision, we found that 48 out of the 96 miRNAs that scored less than 1 returned good BLAST hits to known vertebrate miRNAs in miRBase. The remaining 48 could not be specifically identified, but included 8 in clusters and 13 that had BLAST hits to mature miRNAs in other bats. Our further effort to prioritise novel miRNA candidates based on the combined evidence provides additional guidance to assist with selecting candidates for further study.

One miRNA (pal-can-276) featured noticeably in the analysis. Representing a homolog of miR-541, this *P. alecto* miRNA contained three unique changes in the mature sequence relative to other vertebrates, including two changes within the critical seed region. Amongst its predicted gene targets was *ZFAT*, a zinc-finger protein with roles in cell survival and apoptosis (particularly in immune-related cells) [26], and the TNF-receptor *TNFRSF1B*. In the rat, miR-541 is described as a brain-specific miRNA involved in neuron proliferation and neurite outgrowth via suppression of synapsin I [27], while the corresponding star sequence (which is also unique to *P. alecto* including one difference in the seed region), has been reported to be downregulated in a region of the brain (the spinal dorsal horn) in rats with experimentally-induced neuropathic pain [28]. It is well established that miRNAs play important roles in apoptosis [29–31], and one possibility is that viruses such as HeV block apoptosis via induction of host miRNAs that downregulate pro-apoptotic genes. *P. alecto* miR-541 is a candidate miRNA in such a scenario, however it remains to be experimentally determined as to whether the differences in the sequence of *P. alecto* miR-541 correspond to differences in function between bats and other mammals, or whether it plays any role in apoptosis.

Four other miRNAs, representing homologs of miR-337, miR-377, miR-513c and miR-885 respectively, were also confirmed to have novel seed sequences in *P. alecto*. In humans, miR-513 is located within a cluster (miR-506:514) that is overexpressed in melanoma [32]. In *P. alecto*, the novel miR-513 is also located within a putative cluster containing 14 other miRNAs (Figure 4b), the majority of which are novel. We note, however, that this cluster may be fast-evolving in mammals [33, 34]. MiRNAs in true genomic clusters are not only close together, but are transcribed as a polycistronic transcript from a single promoter, therefore promoter analysis may also be required to confirm genuine polycistronic clusters [35].

Amongst the 30 most significant novel miRNAs identified in *P. alecto* (Table 1), 15 were homologous to known vertebrate miRNAs but contained unique differences, while the remaining 15 could not be aligned to existing miRNAs and may represent unique or highly divergent bat miRNAs. In addition to those already discussed, several miRNAs within this set have significant functional roles in other mammals, some with potential significance to bat biology. We previously showed that in *P. alecto*, *TP53* (p53) and *MDM2* contain mutations in subcellular localization signals required for nucleocytoplasmic shuttling [19]. MiR-215, which plays a role in the *TP53*:*MDM2* interaction [36, 37], is unique in *P. alecto* and is presented in Table 1.

Bats have a concentration of positively selected genes within the oxidative phosphorylation pathway, believed to reflect adaptations necessary for flight-related energy metabolism [38]. Our recent analysis of bat genomes revealed positive selection within the DNA damage response pathway and NF-κB pathway in bats, possibly reflecting compensatory adaptations to tolerate harmful byproducts produced by elevated metabolism [19]. Target prediction found a putative connection between pal-can-300 (miR-874) and *FANCI*, which is involved in DNA repair. Other miRNAs known to be involved in oxidative phosphorylation include miR-34a [39] and miR-338 [40], however both of these are conserved in *P. alecto*.

Future directions involving miRNAs in the Black flying fox include analysis of differential miRNA expression, correlating miRNA expression with that of putative gene targets, and experimental validation of targets for individual miRNA candidates. Such studies will be greatly facilitated by the dataset, methods, and analysis pipeline provided in this study.

## Conclusions

In summary, 399 putative miRNAs have been identified in the Black flying fox small RNA transcriptome. This includes a number of novel miRNAs that represent homologs of known miRNAs with functions relevant to bat biology. These findings will facilitate further studies of gene regulation in bats in relation to their role as virus reservoirs and may shed light on mechanisms underlying bat-specific traits such as flight and longevity.

## Methods

### High-throughput sequencing of small RNAs isolated from Pteropus alecto

Fourteen tissues (bone, brain, heart, kidney, large intestine, small intestine, liver, lung, muscle, lymph nodes, salivary glands, skin, spleen and testes) were harvested from four male Black flying foxes (*P. alecto*) captured in Brisbane, Queensland, Australia. All experiments were approved by the Australian Animal Health Laboratory Animal Ethics Committee. Equal amounts of tissue from each organ were pooled from the four bats and processed using the MirVana miRNA isolation kit (Ambion, Carlsbad, CA) according to the manufacturers’ guidelines. Bat small RNA was prepared for Illumina sequencing as follows: ~7 μg total RNA was size-fractionated by Novex 15% TBE-Urea gel (Invitrogen, Carlsbad, CA) and RNA fragments between 20 and 30 bases in length were isolated. The purified small RNAs were ligated with 5’ adapters (Illumina, San Diego, CA). To remove un-ligated adapters, ligation products were gel purified on Novex 15% TBE-Urea gel. Subsequently, RNA fragments were ligated with 3’ adapters (Illumina). After gel purification on Novex 10% TBE-Urea gel, fragments with adapters at both ends (70–90 bases in length) were reverse transcribed and subjected to 15 cycles of PCR. Amplification products were loaded on Novex 6% TBE gel (Invitrogen) and the gel band containing 90- to 100-bp fragments was excised. Purified cDNA was used directly for cluster generation and 36 cycles of sequencing analysis using the Illumina Cluster Station and 1G Genome Analyzer following manufacturer’s protocols.

### Identification of miRNAs in deep sequencing data

The quality of Illumina deep sequencing data was examined using FastQC (version 0.9.5) [41]. Adapters were trimmed using Cutadapt (version 1.2.1) [42] with the following options: −a TCGTATGCCGTCTTCTGCTTG --discard-untrimmed -m 18 -M 26 -O 10. The option -a indicates the adapter sequence to trim, while -m and -M designate the minimum and maximum length reads to keep after trimming. -O indicates the minimum number of matching bases required to trigger adapter recognition. Trimmed reads were filtered using the FASTX-Toolkit [43]. Fastx_artifacts_filter was run with default settings to remove reads consisting of > 90% homopolymers, followed by fastq_quality_filter with the following options: −q 16 -p 90, meaning at least 90% of bases in a read needed quality scores of at least 16 for the read to be retained). Filtered reads were processed for miRNA content using miRDeep2 [44]. Filtered reads were pre-processed using the mapper.pl module with the following options: −e -j -h -m. The -e option designates FASTQ input data, −j removes sequencing reads with called nucleotides other than A, C, G, T and N, −h converts to FASTA format and -m assigns reads with identical sequence to a single FASTA entry with a header tag designating the combined read count. Pre-processed reads were then analysed for miRNA content using the miRDeep2.pl module with the following options: mature_ref_vertebrates mature_ref_vertebrates none -a 1 -b −5. The first three fields designate reference files containing known miRNAs (mature this species, mature other species, precursor this species). In the absence of known *P. alecto* miRNAs, a database consisting of all known vertebrate mature miRNAs (as recorded in miRBase version 20) was constructed and used in both of the first two fields, while the third field was left intentionally blank (“none”). The option -a indicates the minimum read depth, while -b indicates the minimum miRDeep2 score for a hit to be retained (Note that novel miRNAs with only a single read were later filtered out as described below). All miRNAs identified by miRDeep2 as “known” were retained according to these settings, while “novel” miRNAs were further filtered according to the following rules: Group 1 (score ≥ 1, read depth ≥ 2), or Group 2 (score ≥ −5, read depth ≥ 5). “Novel” miRNAs that did not fall into either category were considered unreliable and were discarded.

### Bioinformatics analysis of bat miRNAs

Overlapping miRNAs, miRNAs within introns, physical clustering and seed sequence usage were analysed using in-house python scripts. Non-coding RNAs (ncRNAs) were identified by RFAM batch search [45]. Homologs were identified using standalone command line BLAST (version 2.2.29) [46]. For comparison, vertebrate mature, star and precursor miRNA sequences were obtained from miRBase (version 20) [47]. The vertebrate reference miRNA set consisted of 38 species, made up of 25 mammals (5 Laurasatherians, 13 primates, 3 rodents, 3 marsupials, 1 monotreme), and 13 non-mammals (10 fish, 2 birds, 1 reptile). *Pteropus vampyrus* and *Myotis lucifugus* predicted miRNA precursor sequences were obtained from Ensembl. Additional datasets were obtained from supplementary materials accompanying published articles by Biggar et al. [9], Platt et al. [13]. For identification of mature and star miRNAs by BLASTN, the following options were used: −strand plus -evalue 10 -word_size 4 -penalty −4 -reward 5 -gapopen 8 -gapextend 6. MiRNAs were then categorised as 100% match, mismatch or no hit, relative to their top BLAST hits. Within the 100% match category, miRNAs were allowed to have up to 3 bp over/underhanging at the ends (a maximum of 2 bp was allowed for each individual end), relative to their top BLAST hit. This step that we refer to as ‘end-anchoring’ was accomplished using the custom BLAST output format and the command-line tool awk, and was deemed necessary to account for minor length variations in mature forms, but to screen out hits in which the alignment is merely truncated due to terminal mismatches. For identification of precursor miRNAs, the same parameters were used, except the minimum e_value was set to 0.001 and hits were filtered for > 80% identity and > 80% overlap using awk. Top hits were identified by e-value from the BLAST output using in-house python scripts. If multiple high-scoring hits were present, hits were further ranked by species in the following order (human, horse, cow, pig, dog, sheep, mouse, rat, macaque, gorilla, other) to facilitate downstream annotation analysis. Multiple sequence alignments were performed using MEGA (version 5) [48]. Putative miRNA targets were identified using miRanda [49] with the following options: −en −20 -strict. The -en option defines the minimum free energy threshold for a miRNA-target pairing, while the -strict option demands perfect complementarity between the miRNA seed region and the target. A database for miR:Target prediction was constructed as follows: Using *P. alecto* transcriptome data assembled against the reference *P. alecto* genome [19], 3’ UTRs were extracted using a python script. Sequences were filtered to exclude those that exceeded 2500 nt in length as they may not reflect genuine mRNAs, while others lacking gene IDs were excluded on the basis that they were unsuitable for annotation enrichment analysis. Functional annotation clustering analyses (GO and KEGG) were performed using the DAVID web service [50] accessed via python scripts. Due to the large number of hits for GO enrichment, an additional filter of FDR < 1.0 was applied.

### Availability of supporting data

The raw sequencing data have been deposited in the Sequence Read Archive (SRA) under BioProject PRJNA210946. MicroRNA sequences have been submitted to miRBase.

## Electronic supplementary material

Additional file 1: Figure S1: FASTQC analysis of raw data quality. Raw data was assessed for overall quality at the outset (A) and after filtering (B) using the FASTQC application. Numbers on the x-axis correspond to base position (only the first 26 bp of raw reads are shown since all reads were trimmed to a maximum of 26 bp during pre-processing), while numbers on the y-axis represent the quality score at each base position. The central red line is the median value, yellow boxes represent the inter-quartile range (25-75%), upper and lower whiskers represent the 10% and 90% points, while the blue line represents the mean quality. The green area corresponds to reads of very good quality, orange to reasonable quality and red to poor quality. (PDF 141 KB)

Additional file 2: Table S1: *P. alecto* miRNAs identified using miRDeep2. MiRDeep2 assigns each predicted miRNA a score that represents its likelihood of being a genuine miRNA. Read counts, sequences (mature, star, precursor), genomic coordinates and statistical test results (significant randfold p-value) are also included in the output. (XLSX 93 KB)

Additional file 3: Table S2: *P. alecto* miRNAs with overlapping genome coordinates Pairs of predicted miRNAs in which the precursors had overlapping genomic coordinates (and in one case, an overlapping triplet) are presented along with their miRDeep2 scores, read counts and relative orientations. It is likely that each pair represents a single miRNA locus, but further validation will be required to establish which of the two (or three) alternatives represents the genuine precursor. Instances in which one member of an overlapping pair scored substantially higher than the other are indicated, and the cause and significance of this is described in the text. In these cases the lower scoring precursor was discarded from the analysis. (XLSX 48 KB)

Additional file 4: Table S3: Homology of *P. alecto* miRNAs to RFAM entries. *P. alecto* miRNA precursors were compared with all known non-coding RNAs in the RFAM database (version 11). While the vast majority of hits were to miRNA families, several bat miRNAs had hits to other categories of non-coding RNA. (XLSX 85 KB)

Additional file 5: Table S4: Homology of *P. alecto* miRNAs to known miRNAs in other vertebrates. BLASTN was used to compare all *P. alecto* miRNAs with all vertebrate miRNAs in the miRBase database (version 20) as well as three sources of bat miRNA sequence data: Biggar et al. [9]; Platt et al. [13]; Ensembl (*P. vampyrus*, *M. lucifugus*). Searches were conducted separately for mature, star and precursor forms of each miRNA, and the best hit kept for each, along with the details of the BLAST hit. Please refer to the materials and methods for details of the search parameters and a description of the best-hit selection method. Mature and star hits were assigned to one of the following categories: 100% match, mismatch or no-hit. Up to 2 overhanging nucleotides (and a maximum of 3 when both ends were taken into account) were allowed at the ends of matches, but mismatches of the terminal bases were not allowed. MiRNAs with under/overhangs but no internal mismatches are included in the 100% category. MiRNA families are indicated where known. (XLSX 237 KB)

Additional file 6: Table S5: *P. alecto* miRNAs located within introns and exons. The genomic coordinates of all *P. alecto* miRNA precursors were compared with those of all annotated protein-coding genes. MiRNAs located fully within introns or exons are indicated, as well as those that lie across exon boundaries. Only miRNAs located on the same strand as their parent gene are reported. (XLSX 56 KB)

Additional file 7: Table S6: Clustering of *P. alecto* miRNAs on genome scaffolds. Genomic coordinates were used to identify clusters of proximal miRNAs within the *P. alecto* genome. An upper limit of 5000 bp was used for cluster identification, and the minimum cluster size was two. MiRNAs with overlapping coordinates were treated as single entities in the cluster analysis as they likely represent only one genuine miRNA, as describe in the text. (XLSX 50 KB)

Additional file 8: Figure S2: Alignment of bat miRNAs forming a highly divergent cluster. Fifteen *P. alecto* miRNAs shown to be probable members of a cluster homologous to the human ChrX miR-506:514 cluster (Figure 4) were aligned using MEGA. While only seven returned BLAST hits to known miRNAs (as indicated in brackets), the majority showed some degree of homology when aligned. (PDF 91 KB)

Additional file 9: Table S7: Analysis of *P. alecto* miRNA seed sequences. The sequence of nucleotides 2–7 for each mature miRNA were examined and compared with the repertoire of seed sequences present in vertebrate mature miRNAs in miRBase version 20. This provides an overview and allows comparison of frequent and rare seed usage in *P. alecto* and the miRBase vertebrate dataset, and illustrates the degree of seed diversity present in *P. alecto* miRNAs. A total of 21 *P. alecto* miRNAs had seed sequences distinct from those in the miRBase vertebrate collection, however additional methods were required to confirm the presence of novel seed sequences, as described in further detail in the text. (XLSX 45 KB)

Additional file 10: Table S8: Putative transcription factor targets of *P. alecto* miRNAs. Target prediction was performed for all *P. alecto* miRNAs using Miranda software and a database constructed of expressed *P. alecto* transcripts. The database consisted of 3’UTRs extracted from a reference-assembled transcriptome, and gene targets corresponding to transcription factors were identified according to a survey of human transcription factors [22]. For each miRNA, its top BLAST hit and per cent identity to that hit are included to facilitate comparison with known miRNAs. (XLSX 40 KB)

Additional file 11: Table S9: GO enrichment of predicted gene targets. Lists of predicted gene targets for each miRNA were subjected to Gene Ontology (GO) enrichment analysis. For each hit, the raw p-value is indicated, as well as Bonferroni, Benjamini and False Discovery Rate (FDR) corrections, to account for multiple hypothesis testing. The background consisted of all named genes present in the 3’UTR database used for target prediction. (XLSX 45 KB)

Additional file 12: Table S10: KEGG enrichment of predicted gene targets. Lists of predicted gene targets for each miRNA were subjected to categorical enrichment analysis for molecular pathways listed in the Kyoto Encyclopaedia of Genes and Genomes (KEGG). For each hit, the raw p-value is indicated, as well as Bonferroni, Benjamini and False Discovery Rate (FDR) corrections, to account for multiple hypothesis testing. The background consisted of all named genes present in the 3’UTR database used for target prediction. (XLSX 106 KB)
