# Supplementary figures and images for: Characterisation of novel microRNAs in the Black flying fox (Pteropus alecto) by deep sequencing
Source: BMC Genomics. 2014 Aug 15;15(1):682. doi: 10.1186/1471-2164-15-682 (PMC4156645; doi:10.1186/1471-2164-15-682)

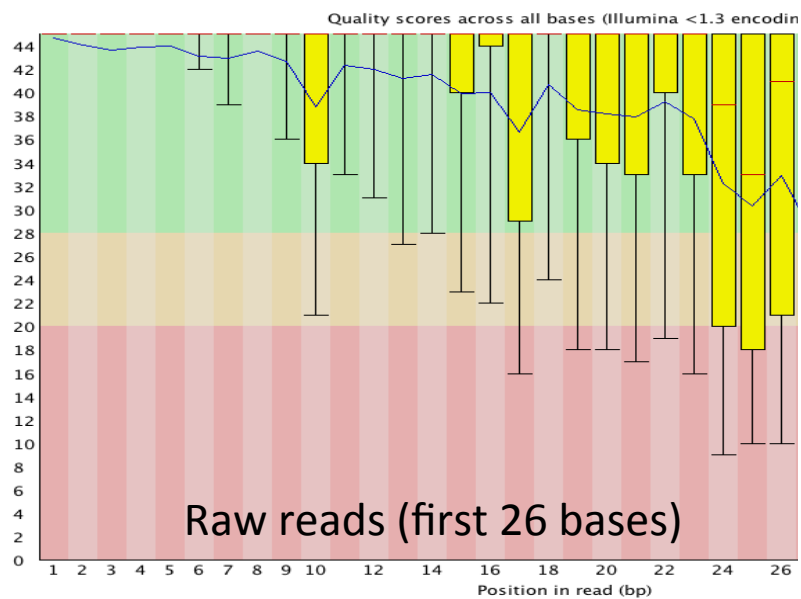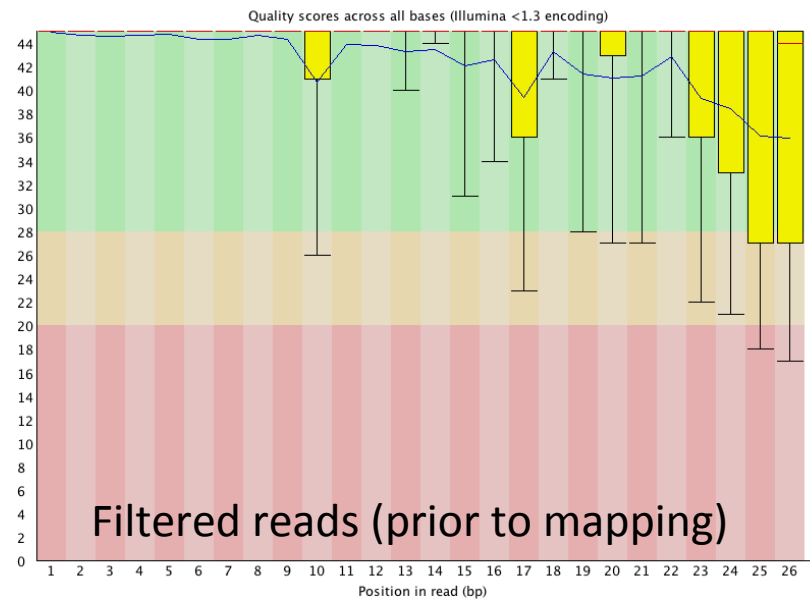

Supplement: Supplementary file 1 — Additional file 1: Figure S1: FASTQC analysis of raw data quality. Raw data was assessed for overall quality at the outset (A) and after filtering (B) using the FASTQC application. Numbers on the x-axis correspond to base position (only the first 26 bp of raw reads are shown since all reads were trimmed to a maximum of 26 bp during pre-processing), while numbers on the y-axis represent the quality score at each base position. The central red line is the median value, yellow boxes represent the inter-quartile range (25-75%), upper and lower whiskers represent the 10% and 90% points, while the blue line represents the mean quality. The green area corresponds to reads of very good quality, orange to reasonable quality and red to poor quality. (PDF 141 KB) [file 12864_2013_6398_MOESM1_ESM.pdf]
