# Supplementary material for: Characterisation of novel microRNAs in the Black flying fox (Pteropus alecto) by deep sequencing
Source: BMC Genomics. 2014 Aug 15;15(1):682. doi: 10.1186/1471-2164-15-682 (PMC4156645; doi:10.1186/1471-2164-15-682)
Supplement: Supplementary file 8 — Additional file 8: Figure S2: Alignment of bat miRNAs forming a highly divergent cluster. Fifteen P. alecto miRNAs shown to be probable members of a cluster homologous to the human ChrX miR-506:514 cluster (Figure 4) were aligned using MEGA. While only seven returned BLAST hits to known miRNAs (as indicated in brackets), the majority showed some degree of homology when aligned. (PDF 91 KB) [file 12864_2013_6398_MOESM8_ESM.pdf]

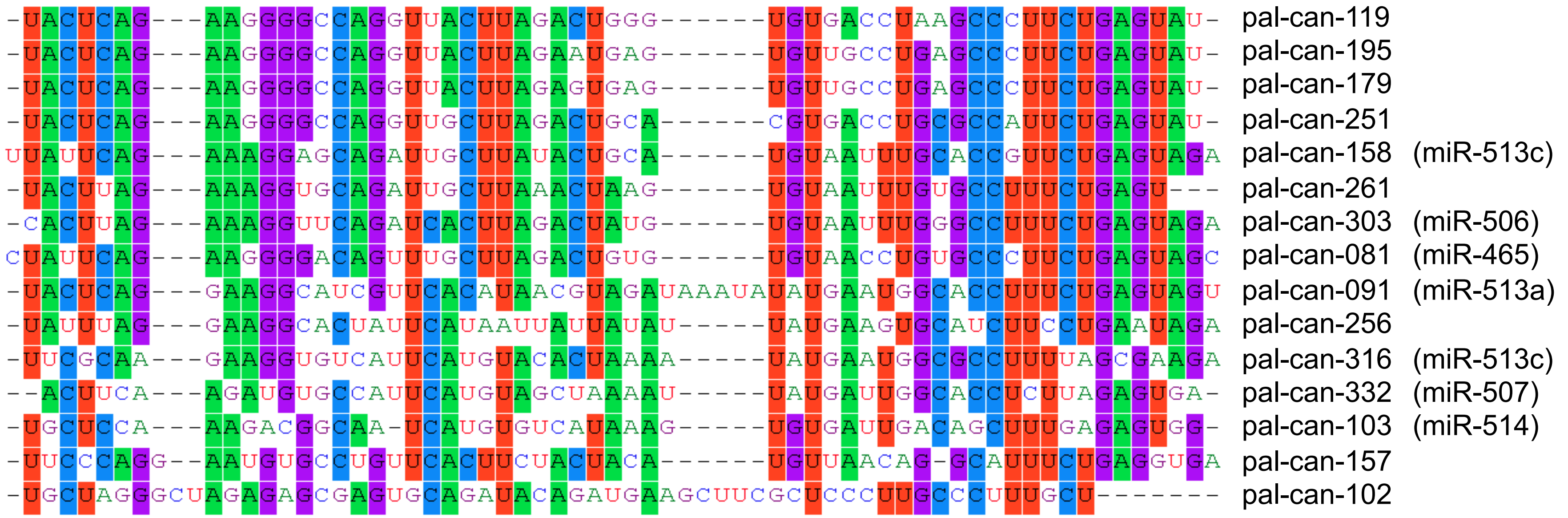
-UACUCAG---AAGGGCCAGGUUACUUAGACUGGG------UGUGACCUAAGCCCUUCUGAGUAU- pal-can-119
-UACUCAG---AAGGGCCAGGUUACUUAGAAUGAG------UGUUGCCUGAGCCCUUCUGAGUAU- pal-can-195
-UACUCAG---AAGGGCCAGGUUACUUAGAGUGAG------UGUUGCCUGAGCCCUUCUGAGUAU- pal-can-179
-UACUCAG---AAGGGCCAGGUUGCUUAGACUGCA------CGUGACCUGCGCCAUUCUGAGUAU- pal-can-251
UUAUUCAG---AAAGGAGCAGAUUGCUUAUACUGCA------UGUAAUUUGCACCGUUCUGAGUAGA pal-can-158 (miR-513c)
-UACUUAG---AAAGGUGCAGAUUGCUUAAACUAAG------UGUAAUUUGUGCCUUUCUGAGU--- pal-can-261
-CACUUAG---AAAGGUUCAGAUCACUUAGACUAUG------UGUAAUUUGGGCCUUUCUGAGUAGA pal-can-303 (miR-506)
CUAUUCAG---AAGGGACAGUUUGCUUAGACUGUG------UGUAACCUGUGCCCUUCUGAGUAGC pal-can-081 (miR-465)
-UACUCAG---GAAGGCAUCGUUCACAUAACGUAGAUAAAUAUAUGAAUGGCACCUUUCUGAGUAGU pal-can-091 (miR-513a)
-UAUUUAG---GAAGGCACUAUUCAUAAUUAUUAUAU-----UAUGAAGUGCAUCUUCCUGAAUAGA pal-can-256
-UUCGCAA---GAAGGUGUCAUUCAUGUACACUAAAA-----UAUGAAUGGCGCCUUUUAGCGAAGA pal-can-316 (miR-513c)
--ACUUCA---AGAUGUGCCAUUCAUGUAGCUAAAAU-----UAUGAUUGGCACCUCUUAGAGUGA- pal-can-332 (miR-507)
-UGCUCCA---AAGACGGCAA-UCAUGUGUCAUAAAG-----UGUGAUUGACAGCUUUGAGAGUGG- pal-can-103 (miR-514)
-UUCCCAGG--AAUGUGCCUGUUCACUUCUACUACA------UGUUAACAG-GCAUUUCUGAGGUGA pal-can-157
-UGCUAGGGCUAGAGAGCGAGUGCAGAUACAGAUGAAGCUUCGCUCCCUUGCCCUUUGCU------- pal-can-102
